# A Data-Driven Approach for Studying the Influence of Carbides on Work Hardening of Steel

**DOI:** 10.3390/ma15030892

**Published:** 2022-01-24

**Authors:** Martina Vittorietti, Javier Hidalgo, Jesús Galán López, Jilt Sietsma, Geurt Jongbloed

**Affiliations:** 1 Department of Applied Mathematics, Faculty EWI, Delft University of Technology, Mekelweg 4, 2628 CD Delft, The Netherlands; G.Jongbloed@tudelft.nl; 2 Materials Innovation Institute (M2i), Mekelweg 4, 2628 CD Delft, The Netherlands; 3 Department of Economics, Business and Statistics, University of Palermo, Viale delle Scienze, Edificio 13, 90133 Palermo, Italy; 4 ETSII-INEI, DYPAM Research Group, Universidad de Castilla La Mancha, Avda. Camilo Jose Cela s/n, 13071 Ciudad Real, Spain; javier.hidalgogarcia@uclm.es; 5 MSE, Faculty 3mE, Delft University of Technology, Mekelweg 2, 2628 CD Delft, The Netherlands; J.GalanLopez@tudelft.nl (J.G.L.); J.Sietsma@tudelft.nl (J.S.)

**Keywords:** synthetic microstructure, stress–strain diagram, FPCA, Voronoi diagrams, Voce law, linear mixed-effects model

## Abstract

This study proposes a new approach to determine phenomenological or physical relations between microstructure features and the mechanical behavior of metals bridging advanced statistics and materials science in a study of the effect of hard precipitates on the hardening of metal alloys. Synthetic microstructures were created using multi-level Voronoi diagrams in order to control microstructure variability and then were used as samples for virtual tensile tests in a full-field crystal plasticity solver. A data-driven model based on Functional Principal Component Analysis (FPCA) was confronted with the classical Voce law for the description of uniaxial tensile curves of synthetic AISI 420 steel microstructures consisting of a ferritic matrix and increasing volume fractions of $M_{23}C_{6}$ carbides. The parameters of the two models were interpreted in terms of carbide volume fractions and texture using linear mixed-effects models.

## 1. Introduction

Describing mechanical properties of macroscopic materials in relation to their microstructural features has been the focus of many studies. Multiple attempts to formulate physics-based and phenomenological models can be found in the literature [1,2,3,4,5,6].

The high number of microstructural parameters, the interrelation among them, and external factors, such as measurement accuracy and limited experimental control, make the identification of a clear relation between microstructure features and mechanical properties, such as material strengthening, very hard to achieve. Accounting for multiple physical factors increases reliability but often results in complex models requiring a trustworthy acquisition of parameters. Phenomenological models are popular due to their simplicity, but they typically lack a clear physics-based interpretation of their parameters. These parameters can be related to microstructure features by designing appropriate experiments that isolate the effect of specific variables. However, controlling microstructures to identify certain relations is a very ambitious goal in real experiments.

The relationship between the volume fraction of carbides and the flow stress has been the subject of several studies [7,8,9,10]. However, as claimed in [11], it is hard to experimentally separate the precipitation contribution to the work-hardening due to the complex nature of steel microstructures. The use of synthetic microstructures in combination with statistics tools may help to formulate an alternative. This concept was explored in this work by introducing a new statistical modeling approach.

As a proof of concept, this technique was applied to the analysis of the effect of $M_{23}C_{6}$ carbide volume fraction on the uniaxial tensile behaviour of AISI 420 steel.

The first step is the synthetic generation of microstructures. An appropriate model for representing steel microstructures needs to be selected. The advantage of the generation of model microstructure is the possibility of building microstructures with a complete control of their morphology that will allow the identification of specific relations between the microstructure features and the mechanical behavior of the material. In [12], a very extensive review on the representation of the morphology of microstructures is presented.

In the present work, the synthetic generation of microstructures was based on matching (or partially matching) statistical and stereological parameters of the real microstructures [13].

Methods like Voronoi tessellations [14], cellular automata [15], and Monte Carlo Potts models [16] are commonly applied for the generation of virtual microstructures. Specifically, Voronoi tessellations are at the forefront of the geometrical models used for representing polycrystalline microstructures, since the growth process of their cells mimics the growth of grains after nucleation [6,17]. Even the most basic case, the Poisson–Voronoi diagram, has shown its power in approximating single-phase microstructures [12]. For more complex microstructures, models such as controlled-Voronoi diagrams [6,18], Laguerre-Voronoi diagrams [19], and multi-level Voronoi diagrams [20,21] have been proposed. In this study, the latter model was considered. One of the advantages of using multi-level Voronoi diagrams is the possibility of including grains with a variety of size and shape distributions of different phases or precipitates, making the resulting synthetic microstructure more realistic.

Seventy synthetic microstructures with an increasing carbide volume fraction and different texture were generated. More specifically, ten different randomly generated crystallographic textures were considered, and, for each of these configurations, seven microstructures with identical geometrical properties but different carbide volume fractions were generated. The idea behind this computational design, called a randomized block design, is to prevent the rise of “spurious relations” due to the well known influence of the orientation on the strain development [22]. After the generation of synthetic microstructures, simulations of the mechanical behavior of the material were performed. For this purpose, the Düsseldorf Advanced Material Simulation Toolkit (DAMASK) [4] was used. DAMASK combines a crystal plasticity formulation with finite-element and spectral solvers, allowing for instance to perform large scale simulations [23], to model damage or fracture [24] or to reproduce the local strain development under uniaxial tensile deformation [25].

In material strengthening simulations, via uniaxal tensile testing, the main results are stress–strain curves, in this case corresponding to microstructures with different carbide volume fractions and textures. For understanding the influence of these microstructural parameters on the stress–strain curve, two different approaches were considered. The first is to study the influence of the microstructural features on the parameters of the Voce model [26]. The Voce model has proven its validity in modeling the plastic deformation of materials with different carbon content [27]. The second approach is a new data-driven approach based on functional data analysis. The stress–strain curves are treated as realizations of a functional model, and their variability is studied using Functional Principal Component Analysis (FPCA). Although the use of principal component analysis and of the functional extension of the method, FPCA, is not very common in materials science, some interesting applications can be found in [28,29]. FPCA is one of the most common tools in functional data analysis, used for understanding the different sources of variability among functions [30].

For interpretation purposes, a modified version of the classical approach was proposed: the functions are not centered on the mean of all curves but on the expected stress–strain curve one would observe for a microstructure without carbides.

For both approaches, the dependence of the model parameters on the carbides volume fraction is explained using linear mixed-effects models [31]. The advantage of using mixed-effects models is the possibility of respecting the hierarchical structure of the data [32]. In fact, the primary interest is on the effect of the carbide volume fraction, but the texture can be responsible for a variance increase and therefore needs to be taken into account as well.

Finally, the validity of the results was evaluated qualitatively comparing the approaches.

The article outline is the following. In Section 2.1, the synthetic microstructure generation using the multi-level Voronoi diagram is presented. The experimental design and the choice of the microstructural parameters are described and discussed. A brief analysis of textures is presented.

After introducing the crystal plasticity software and the advantages of using simulations, the results of the simulated mechanical behaviour of the different microstructures obtained via DAMASK are shown in Section 2.2. In Section 2.3, after having reviewed the basic model for the tensile behaviour of steels, the stress–strain curves corresponding to the different synthetic microstructures are statistically analyzed: in Section 2.4, the results for the Voce model are reported; in Section 2.5, the functional data analysis approach is introduced, and the results are shown. Linear mixed-effects models for interpreting the model parameters in terms of the carbide volume fraction and the texture are introduced in Section 2.6. Interpretation of the results and goodness of fit are discussed in Section 3. The results and final considerations are discussed in Section 4, and conclusions are presented in Section 5.

## 2. Materials and Methods

### 2.1. Synthetic Microstructures Generation

This section describes the generation of synthetic microstructures containing geometrical, physical, and mechanical information in agreement with the microstructure features of the materials to study. The synthetic microstructures generated in this work aim to represent typical annealed AISI 420 steel microstructures containing increasing volume fractions of coarse $M_{23}C_{6}$ carbides embedded in a ferritic matrix. Geometrical and mechanical information of the material used are given and validated in a previous study [25].

For studying the influence of carbides volume fraction on work hardening, taking into account the variation caused by the texture, a randomized block experiment with two classification factors was set. During deformation the grains in a polycrystalline material tend to rotate in relation to the loading mode [33], and therefore specific initial texture conditions can lead to different mechanical responses. In randomized block experiments, the two factors have different roles and importance: one factor is called the blocking factor and it represents a known source of variability in the experiment; the other is called the experimental factor, and the purpose of the study is to determine if there are systematic differences with respect to its values [31]. In this specific case, the texture is the blocking factor, and it defines ten different blocks, each one containing seven synthetic microstructures homogeneous in all their geometrical, chemical, and physical properties except for the levels of the experimental factor, the carbides’ volume fraction.

First, the geometrical structure underlying the microstructure was created. The multi-level Voronoi diagram was selected as a model for representing the microstructures. As previously mentioned, the advantage of using multi-level Voronoi diagrams is the possibility of accounting for complex microstructures, including non-convex grains and different grain-size and shape distributions for grains of different phases or precipitates. The idea behind the construction of multi-level Voronoi diagrams is to stack layers of tessellation with decreasing intensity parameters of the generator points. Without loss of generality we introduce the multi-level Voronoi diagrams considering two tessellation layers.

Consider two finite sets of distinct points in $R^{d}$, $X_{1}=\left\{x_{k1}:k=1,\ldots n\right\}$ and $X_{2}=\left\{x_{i2}:i=1,\ldots m\right\}$ and m<n. Here, $\left\{x_{k1}\right\}$ are the generator points of the first-level tessellation, and the first-level cells are defined as

(1) $$C_{k1}=\{y\in R^{d}:||x_{j1}-y||\leq \left|\right|x_{k1}-y||,j\neq k\},\;\;\;k=1,\ldots ,n.$$

Here ||·|| is the usual Euclidean norm. Let for all $x_{i2}\in X_{2}$

(2) $$I_{i}=\left\{k\in \left(1,\ldots ,n\right):|\right|x_{i2}-x_{k1}\left|\right|\leq \left|\right|x_{i2}-x_{l1}||,l\neq k\},\;\;\;i=1,\ldots ,m,$$

be a set of indices. The cells of the resulting multi-level Voronoi tessellation, also referred as second-level cells or grains, are given by:

(3) $$C_{i}^{*}=\underset{k\in I_{1}}{⋃}C_{k1},\;\;\;\;\;i=1,\ldots ,m.$$

Loosely speaking, given the two point sets $X_{1}$ and $X_{2}$ (m<n), all first-level cells are merged if their generator points are nearest to the second generator point with respect to all others. If the sites of both the first and the second tessellation are generated according to Poisson processes, $\Phi_{1}$ and $\Phi_{2}$, with intensity parameters $\lambda_{1}$ and $\lambda_{2}$, respectively ($\lambda_{1}>\lambda_{2}$), we refer to the resulting tessellation as a multi-level Poisson–Voronoi diagram, ${\mathrm{MV}}_{\left(\Phi_{1},\Phi_{2}\right)}$.

However, unlike the case of the tratidional (one-level) Poisson–Voronoi diagrams, there is not a single parameter (the intensity parameter of the underlying Poisson process) determining the distribution of the geometrical characteristics of the grains. In fact, at least two intensity parameters are now responsible for the resulting grain geometry and morphology. Taking values of $\lambda_{1}$ relatively low (satisfying $\lambda_{1}>\lambda_{2}$) results in a tessellation with non-convex grains and with irregular boundaries. As $\lambda_{1}$ increases (limiting case $\lambda_{1}\to \infty$), the resulting diagram approaches to the one-level Poisson–Voronoi diagram based on the second-level generating points, and therefore the resulting grains are convex.

For the construction of synthetic microstructures, the values of the intensity parameters of the first- and second-level cells are chosen to approximately match the experimental values of the volume fractions and of the mean grain size of the material under study. For representing the annealed AISI 420 stainless steel with $M_{23}C_{6}$ carbides (the AISI 420 steel used in this study contains 0.32 wt.% C, 0.2 wt.% Si, 0.3 wt.% Mn, and 13.7 wt.% Cr), $\lambda_{2}=0.5$ and $\lambda_{1}=3$, which means that the ferrite grains and the carbides precipitates of the synthetic microstructures have mean volumes equal to 2 $\mu m^{3}$ and 0.33 $\mu m^{3}$, respectively. The values of the main geometrical features of 1000 grains of the AISI 420 sample used for creating the synthetic microstructures are shown in Table 1.

For representing the two-phase nature of the material, the intensity parameter of the first-level tessellation needs to take into account the volume fractions of the ferrite grains and of the carbides. Therefore, the intensity of the first-level cells is decomposed as:

$$\lambda_{1}=\lambda_{1}^{f}+\lambda_{1}^{c},$$

where $\lambda_{1}^{f}$ is the intensity of the ferrite phase grains, and $\lambda_{1}^{c}$ is the intensity of the carbides. Considering that the observed volume fraction of carbides in stainless steels is usually between 0.03 and 0.11 [25,34], seven different values were considered for $\lambda_{1}^{c}$, namely: 0, 0.01, 0.03, 0.05, 0.07, 0.09, and 0.11; since the value of $\lambda_{1}$ is fixed, $\lambda_{1}^{f}$ changes accordingly.

**Figure 1:**
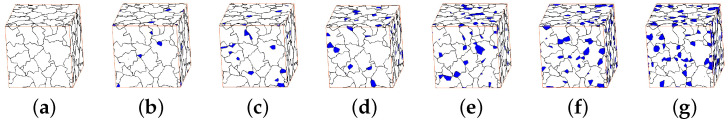
3D multi-level Voronoi diagrams with increasing level of $\lambda_{1}^{c}$: (**a**) $\lambda_{1}^{c}=0$, (**b**) $\lambda_{1}^{c}=0.01$, (**c**) $\lambda_{1}^{c}=0.03$, (**d**) $\lambda_{1}^{c}=0.05$, (**e**) $\lambda_{1}^{c}=0.07$, (**f**) $\lambda_{1}^{c}=0.09$, (**g**) $\lambda_{1}^{c}=0.11$.

The geometry of the synthetic microstructures is shown in Figure 1.

**Figure 2:**
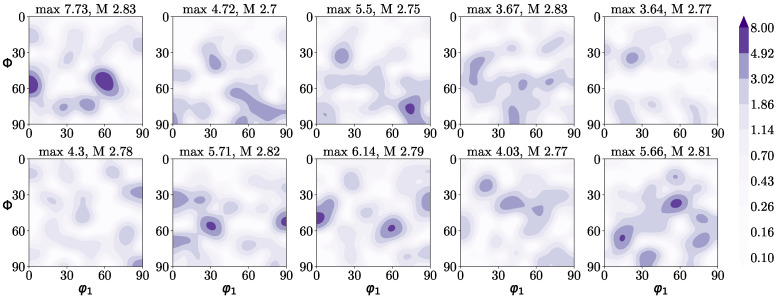
ODFs ($\varphi_{2}=45^{\circ }$ ODF sections) corresponding to each of the ten different crystallographic textures in the ferrite phase under study. An orthorhombic sample symmetry was assumed. Above each texture, it is shown the maximum texture intensity in random units and the Taylor factor calculated using the full-constraints Taylor model (*M*).

This exact same generation was repeated for each of the ten blocks given by the different textures; hence, in total, 70 microstructures were considered. Whereas the ferrite grain size was kept steady, not changing the value of the intensity of the second-level grains, the orientations of the grains were randomly assigned. The ten diverse randomly produced textures are shown in Figure 2.

More specifically, Figure 2 shows the ten $\varphi_{2}=45^{\circ }$ planes of the orientation distribution functions (ODFs) corresponding to each of the generated carbides-free RVEs (Figure 1a). Although orientations were randomly generated, the number of grains in the RVE was relatively small (50 grains), and therefore the obtained texture deviates from what is commonly known as a *random texture*. The maximum intensity value varied from approximately 3.5 to 8 random units. The deviation between these textures was assumed to be reasonable for the range of variation observed in real materials. Figure 2 also shows the Taylor factor, *M*, calculated for each texture using a full-constraints (FC) Taylor model [35]. The Taylor factor represents the degree of plastic shear in the material with respect to the applied macroscopic strain and can be used to estimate the texture effect in macroscopic mechanical behavior. For individual BCC grains, the calculated Taylor factor ranges from 1.78 to 3.71, while the factor calculated using the FC model for an ideal random texture is in BCC metals 2.71 [36], which is relatively close to the values shown in Figure 2.

### 2.2. Virtual Tensile Test

Once the synthetic microstructures are ready, the second step is to perform the virtual experiments. The uniaxial strain and stress development in the different synthetic microstructures is simulated integrating a crystal plasticity model and a spectral solver based on the Fast Fourier Transform (FFT) implemented in the DAMASK software (Düsseldorf Advanced Material Simulation Toolkit [4]). A thorough description of the model and settings used in simulations can be found in [4,25]. The stress in the reversible-strain regime depends only on the elastic strain expressed as the Green–Lagrange strain tensor and the material’s specific stiffness. A hardening law based on the viscoplastic formulation of [37] was implemented to describe the irreversible part of the tensile curve. Parameters for AISI 420 ferrite and $M_{23}C_{6}$ carbides defined in the model constitutive equations can be found in Table 2. Validation of the parameters has been performed in [25]. The values $C_{ii}$ are components of the elastic stiffness tensor; ${\dot{\gamma }}_{0}$ is the initial shear rate; $n_{slip}$ is related to the material’s sensitivity to strain rate; $\tau_{C}^{\eta }$ is the critical shear for stress flow for the slip plane η; $\tau_{sat}$ is the saturation shear stress; $h_{0}$ is the initial hardening; and *a* is a dimensionless parameter related to the material’s hardening.

**Figure 3:**
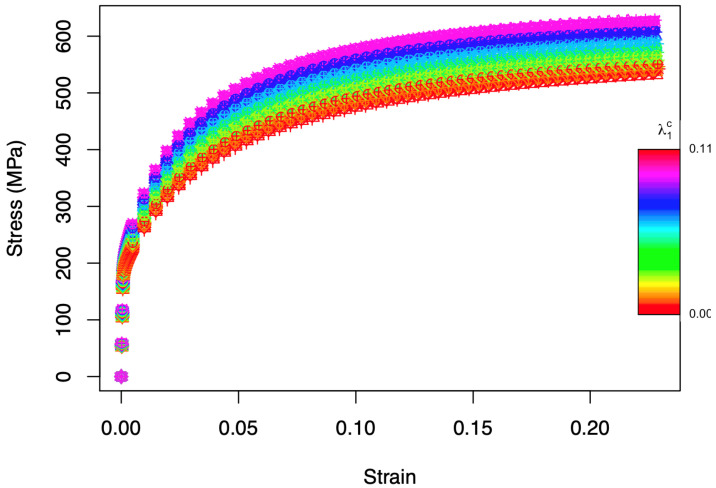
Stress–strain curves. Different colors indicate different values of the volume fraction of the carbides in the range [0, 0.11], with different symbols indicating different textures in the ferrite phase.

A longitudinal strain rate of 0.0001 $s^{-1}$ under uniaxial conditions was imposed in all cases. In Figure 3, the stress–strain curves corresponding to the 70 different microstructures are shown. The color code represents the carbide volume fraction ($\lambda_{1}^{c}$). The deformation regime and hardening behavior of the different curves vary with the strain. The resulting strain–stress curves are clearly affected by the carbide volume fraction and less by the texture that slightly influences the variability within microstructures with a certain carbide fraction. In fact, it is not easy to distinguish the different curves associated to the different textures in the ferrite phase (Figure 3). The influence of carbide volume fraction and texture on strain–stress curves is further investigated in the following sections.

### 2.3. Statistical Analysis

Two approaches were proposed and assessed in this study to relate microstructure features of the artificial microstructures shown in Figure 1 to the functional responses shown in Figure 3: (1) a parametric model based on a mathematical law and (2) a data-driven model based on functional principal component analysis. The formulation of the first approach requires the selection of a law. The strain-hardening response of a material is typically studied using true stress–true-strain curves, as obtained from uniaxial tensile tests. This section revisits some of the models most commonly used to describe the tensile curves of metals.

The total strain ε is decomposed into elastic and plastic strain:

(4) $$\varepsilon =\varepsilon_{e}+\varepsilon_{p}.$$

While elastic strain will be recovered as the material is unloaded, plastic strain is permanent. The elastic component $\varepsilon_{e}$ is proportional to the stress σ, following the well-known Hooke’s Law [38]:

(5) $$\varepsilon_{e}=\sigma /E$$

where *E* is called the elastic or Young’s modulus. However, there is no consensus on how to describe the plastic component $\varepsilon_{p}$ with a single mathematical expression. As the material is subjected to plastic deformation, it will become stronger as a result of work hardening, and the stress required to apply further plastic deformation will increase. Many expressions have been proposed to describe this behavior. In general, the plastic deformation part of the stress–strain curve of several metals can be represented by a power-curve relation [39]:

(6) $$\sigma -\sigma_{y}=K\cdot \varepsilon_{p}^{n}$$

where *K* is the strength coefficient and *n* the strain hardening exponent. Equation (6) is also known as the Hollomon equation [40]. The Hollomon equation is one of the most widely accepted for representing the plastic part of the stress–strain diagram. However, especially for stainless steels, the use of the Hollomon equation is not always recommended [41].

Another commonly used expression, proposed by Ludwik [42], is:

(7) $$\sigma =\sigma_{0}+L\cdot \varepsilon_{p}^{q}$$

where $\sigma_{0}$ is the friction stress, and *L* and *q* are material constants. However, this expression is found not to be appropriate for austenitic stainless steel [41]. A modified version of Equation (6), usually accepted for austenitic stainless steels and in the presence of carbide precipitates [41], was proposed by Ludwigson [43]:

(8) $$\sigma =k_{1}\varepsilon_{p}^{n_{1}}+\exp\left(k_{2}+n_{2}\epsilon_{p}\right),$$

where $k_{1}$, $n_{1}$, $k_{2}$, and $n_{2}$ are material parameters. Modified versions of the Hollomon and Ludwigson equations [27] have also been proposed for metals with different carbon contents. In fact, carbon content is known to be one of the primary factors influencing the strain hardening [27,44].

Voce also proposed a model to describe the plastic flow of metals [26]. The Voce law provides a phenomenological description of the hardening effect produced by the accumulation of plastic deformation, but it is also possible to give a physical interpretation to its parameters [45]. In its simplest form, the Voce law determines the plastic flow of the material in terms of only three parameters. This law has the problem that the stress stabilizes at a certain level of deformation, and this behavior is not commonly observed in metallic materials. In [46], a modified version of the original Voce law was proposed, in which the addition of a fourth parameter allows for an asymptotic hardening rate at large strains. This *extended Voce law* takes the form:

(9) $$\sigma =\tau_{0}+\left(\tau_{1}+\theta_{1}\varepsilon_{p}\right)\left[1-\exp\left(-\varepsilon_{p}\theta_{0}/\tau_{1}\right)\right].$$

In the classical law, $\theta_{1}$ is zero.

However, describing the stress response to strain deformation over the different stages of the tensile test and for a whole range of materials with a single expression is hardly possible [27,47]. In this study, the focus is not only on the identification of a good model for describing the whole range stress–strain behavior but mainly on understanding how the carbide volume fraction influences it. All the described models are potential candidates for such purposes. We selected as a phenomenological model the extended Voce model, and we compared the results with a data-driven model.

### 2.4. Voce Model

**Figure 4:**
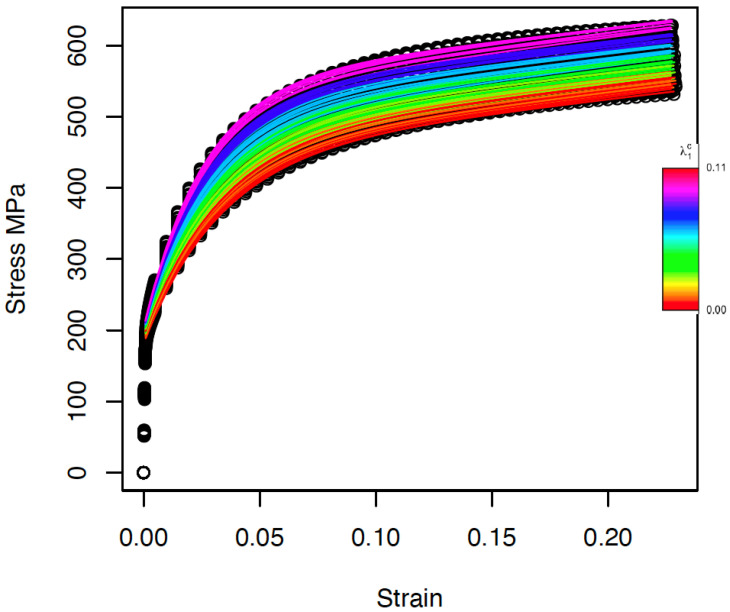
Fitted stress–strain functions using Voce hardening law. Different colors indicate different values of the volume fraction of the carbides in the range [0, 0.11].

For estimating the parameters of the Voce model described in Equation (9), a nonlinear weighted least-squares approach was used [48]. The fitted curves are displayed in Figure 4.

Although some small deviations were observed around a strain of 10%, in general, the model is capable of reproducing the curves simulated by DAMASK with good accuracy. A measure of the goodness of fit is reported in Section 3.1.

### 2.5. Data-Driven Approach

In this section, the influence of the carbides volume fraction on the resulting stress–strain curves (Figure 3) is described in a functional framework. Studying the stress–strain data obtained from the digital experiment as functional data, the aim was to identify an underlying function that can describe the general stress–strain curve for stainless steel and explain its variability in terms of carbide volume fraction. A common approach to represent functional data is assuming an expansion of each sample curve in terms of a linear combination of basis functions [49]. In the most-common settings the basis functions are fixed in advance, and then the coefficients need to be estimated as the main step. Functional Principal Component Analysis (FPCA) constitutes an alternative approach in which the basis functions are estimated in the process.

Principal Component Analysis and its functional extension have been successfully used for data complexity reduction and variability interpretation [30]. The idea is that a function $X_{i}\left(t\right)$ can be expressed in terms of the following expansion:

(10) $$X_{i}\left(t\right)=\mu \left(t\right)+\sum_{k=1}^{\infty }A_{ik}\phi_{k}\left(t\right),$$

or approximately (by truncation) as

(11) $$X_{iK}\left(t\right)\approx \mu \left(t\right)+\sum_{k=1}^{K}A_{ik}\phi_{k}\left(t\right)$$

In the conventional FPCA, μ(t) is the functional mean, obtained as the mean of all the functions $X_{i}\left(t\right)$; $\phi_{k}\left(t\right)$ are the orthonormal eigenfunctions obtained from the spectral decomposition of the covariance function Γ(t,s); and $A_{ik}=\int \left(X_{i}\left(t\right)-\mu \left(t\right)\right)\phi_{k}\left(t\right)dt$ are called the Functional Principal Component Scores. A more-detailed description of the method can be found in [30]. FPCA attempts to find the dominant modes of variation around an overall trend function [50]. In this case, the assumption is that the overall trend is represented by the stress–strain curve for the microstructure without carbides and that the distance from this baseline function is due to the increasing carbide volume fraction and the different texture. A slight modification of the FPCA approach was proposed in this context. The difference with the traditional approach is in the centering of the functions. More precisely, the functions are not centered on the functional mean but on the expected stress–strain curve one would observe for a metal microstructure without carbides. The modified principal component decomposition is:

(12) $$X_{iK}\left(t\right)\approx \mu_{0}\left(t\right)+\sum_{k=1}^{K}A_{ik}\phi_{k}\left(t\right),$$

and

(13) $$A_{ik}=\int \left(X_{i}\left(t\right)-\mu_{0}\left(t\right)\right)\phi_{k}\left(t\right)dt$$

are the modified functional principal components scores. The scores of individual curves on the main eigenfunctions can be used for description, clustering, classification, and prediction [51].

**Figure 5:**
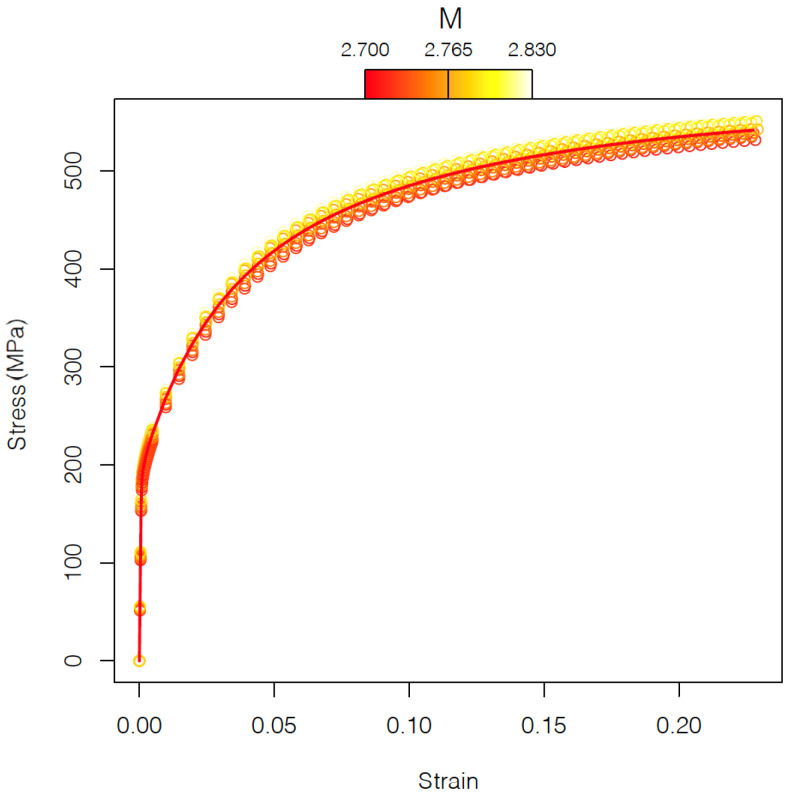
Mean stress–strain curve for microstructures without carbides (red line). Different colors indicate different textures in the ferrite phase with different Taylor factor *M*.

The first step for the modified FPCA approach is defining the mean stress–strain curve for microstructures without carbides. Using the stress–strain values obtained for the ten different microstructures corresponding to the ten different textures, the mean stress–strain curve for the microstructure without carbides is defined as:

(14) $${\hat{\sigma }}_{0}\left(\epsilon \right)=\frac{1}{10}\sum_{j=1}^{10}\sigma_{j0}\left(\epsilon \right),$$

where $\sigma_{j0}$ is the stress–strain curve corresponding to *j*-th texture. In Figure 5, the ten different stress–strain curves corresponding to the microstructures without carbides and the mean stress–strain curve (red line) are shown. The gradient color is based on the Taylor factor *M*.

**Figure 6:**
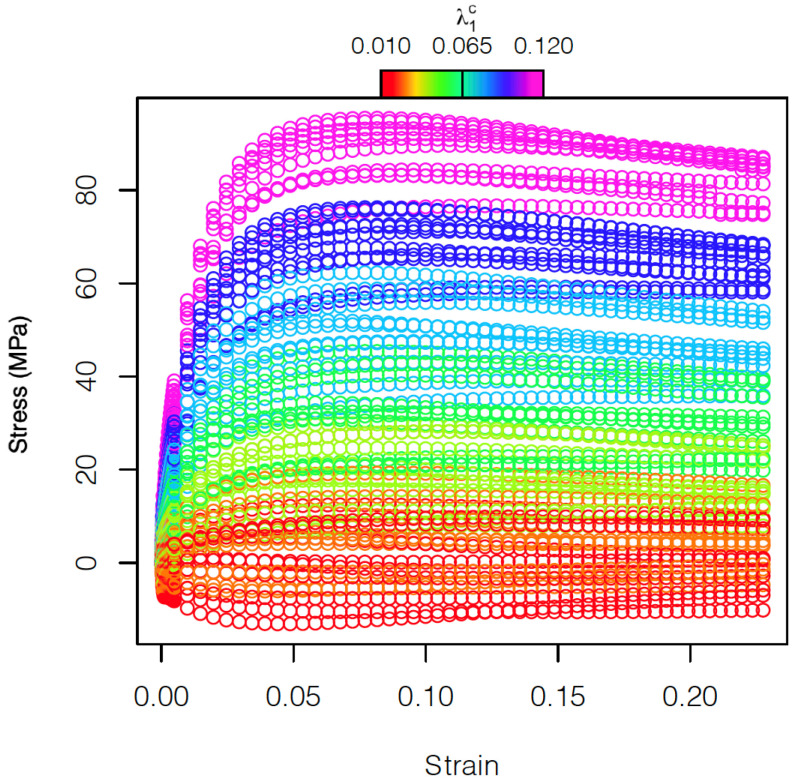
Stress–strain centered to the expected stress–strain for microstructures without carbides. Different colors indicate different values of the volume fraction of the carbides in the range [0, 0.11].

Secondly, the original stress–strain data are centered to the mean stress–strain curve for microstructures without carbides as shown in Figure 6.

**Figure 7:**
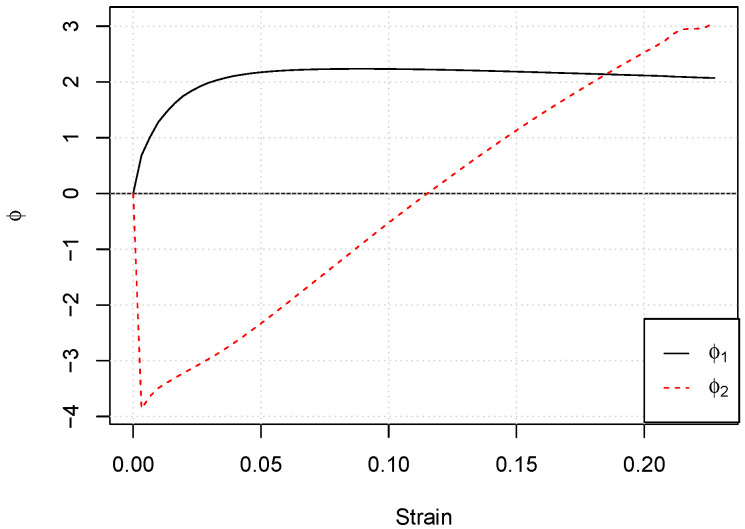
First two eigenfunctions obtained with the modified FPCA performed on the 70 stress–strain curves.

Then, we can perform the modified FPCA. The first two eigenfunctions are plotted in Figure 7. Looking at the behavior of the first eigenfunction $\phi_{1}$, for low strain levels corresponding to the elastic part of the curve, the variance among the curves was low; it reached its maximum around 0.05 strain, and then it slightly decreased. The interpretation of the second eigenfunction $\phi_{2}$ was less intuitive. As the second principal component must be orthogonal to the first one, it defined a less-important mode of variation. It accounted for 2.0% of the total variation and consisted of a high negative contribution for the very low strain values followed by a high positive contribution correspondent to high strain values.

**Figure 8:**
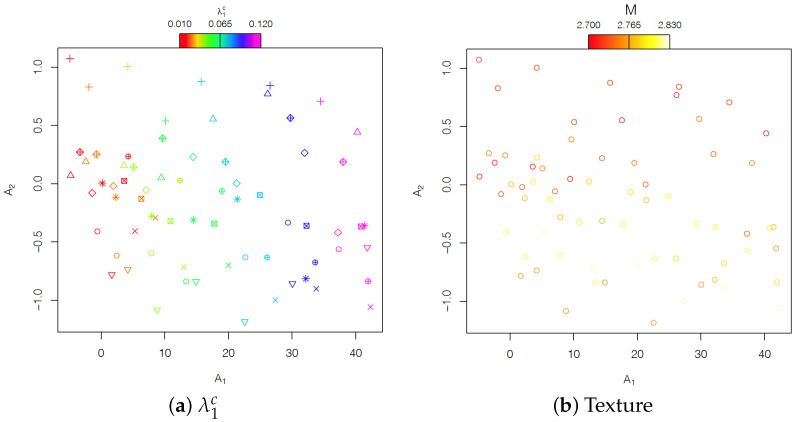
Plot of the two FPCA scores obtained for the 70 stress–strain curves. Different co lours indicate in (**a**) different values of the carbides’ volume fraction in the range [0, 0.11] and in (**b**) different textures in the ferrite phase.

From the plot of the two FPCA scores, $A_{1}$ and $A_{2}$ (Figure 8), some additional considerations can be drawn. Figure 8a shows that one could interpret $A_{1}$ as the effect of the different carbide volume fraction, while Figure 8b shows that $A_{2}$ corresponds to the effect of the different random textures.

**Figure 9:**
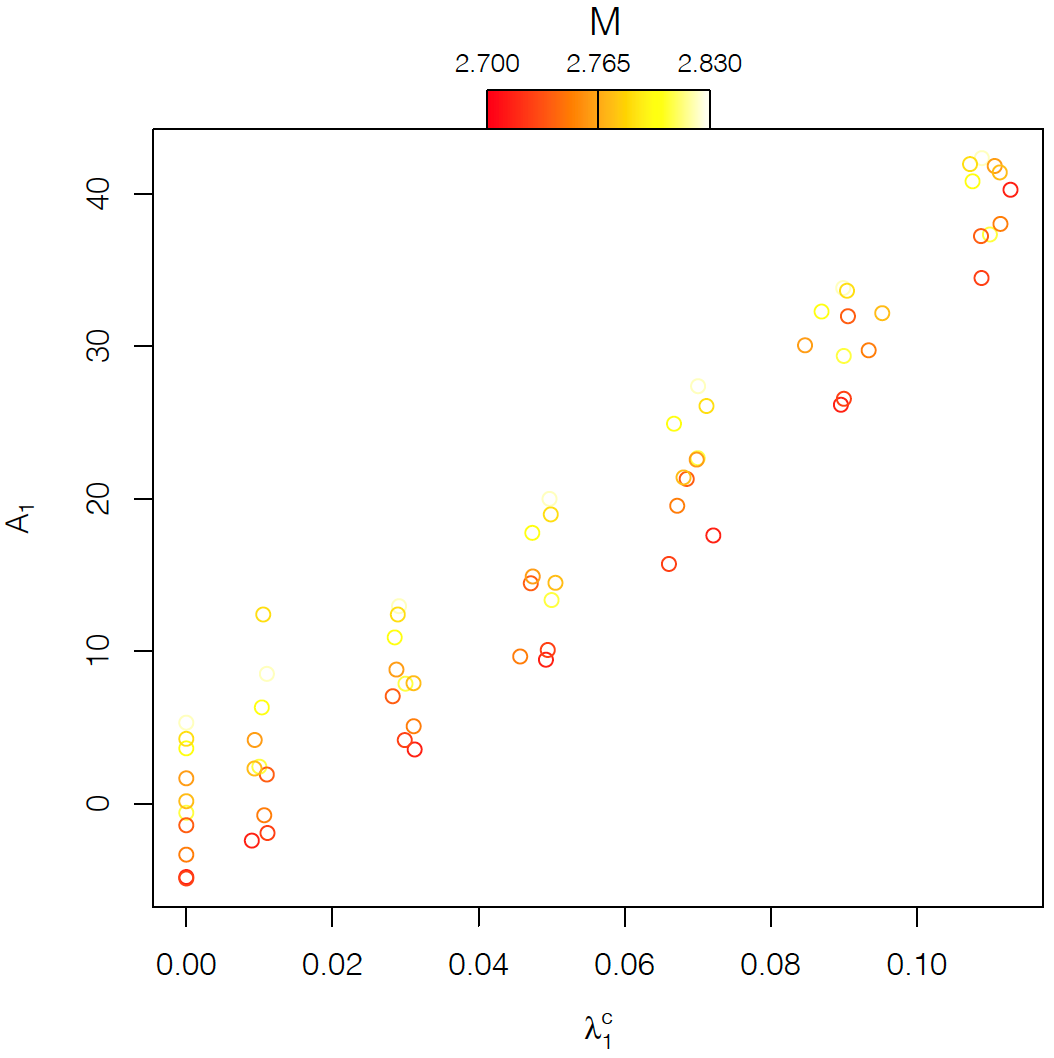
Plot of the FPCA scores correspondent to the first functional principal component $\phi_{1}$ and the observed values of carbide volume fraction $\lambda_{1}^{c}$ for the 70 stress–strain curves. Different colors indicate different textures in the ferrite phase.

One of the aims of FPCA is a reduction in the model complexity. Therefore, given that the first functional principal component explains more than 98% of the total variance around the expected stress–strain curve for the microstructure without carbides, the analysis was reduced to just this component (see Figure 9).

**Figure 10:**
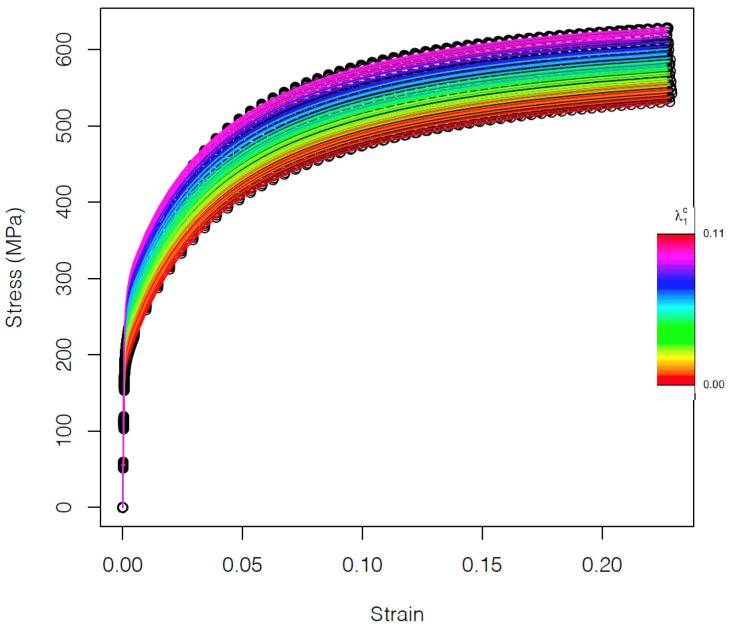
Fitted stress–strain functions using FPCA model (Equation (18)). Different colors indicate different values of the the carbides in the range [0, 0.11].

The results of the model are shown in Figure 10.

### 2.6. Linear Mixed-Effects Model

In this section, linear mixed-effects models are used for expressing the two different model parameters in terms of the carbide volume fraction and the texture. Mixed-effects models are a class of models especially used when the data present a clustered or grouped structure. They are employed to describe the relation between a response variable and the explanatory variables, giving a different role to the classification factors. Two effects were considered:

**Fixed effects**, which concern parameters associated with the levels of the experimental factor or of the explanatory variable whose effect needs to be primarily investigated;
**Random effects**, which concern parameters associated with the levels of the blocking factor or better associated with individuals or groups drawn at random from a population.

Let $y_{i}=\left[y_{i1},\ldots ,y_{in_{i}}\right]$ denote the vector of responses in the *i*-th cluster, i=1,…,k; $X_{i}$ denotes the matrix of explanatory variables for which fixed effects are assumed; let $\beta_{i}$ be the corresponding vector of fixed parameters; $Z_{i}$ denotes the matrix of explanatory variables for which the random effect is assumed; and let $\alpha_{i}$ be the corresponding vector of random parameters. We considered a linear mixed model that assumes heterogeneity of the intercepts only. The model assumptions are:

- $y_{i}|\left(X_{i},1\alpha_{0i}\right)∼N_{n_{i}}\left(X_{i}\beta ,1\alpha_{0i},\sigma^{2}I_{n_{i}}\right)$
- $\alpha_{0i}∼N\left(0,\sigma_{\alpha }^{2}\right)$,

where $\alpha_{0i}$ represents the vector of random intercepts. The model formula is:

(15) $$\mu_{i}=\beta_{0}+\alpha_{0i}+\beta_{1}x_{i},$$

with $\mu_{i}$ indicating the expected response.

## 3. Results

**Figure 11:**
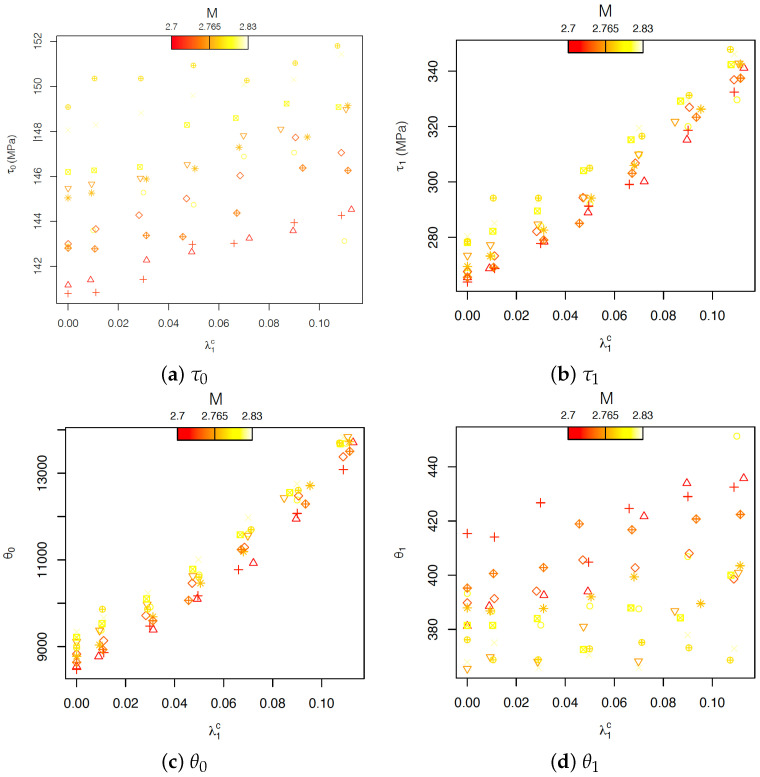
Plot of the estimated Voce-model parameters ($\tau_{0}$ (**a**), $\tau_{1}$ (**b**), $\theta_{0}$ (**c**), $\theta_{1}$ (**d**)) and the observed volume fraction of carbides for the 70 different microstructures (different symbols indicate different textures in the ferrite phase).

Four different linear mixed models (15) were used for evaluating the relationship between the parameters of the Voce model and the carbide volume fraction and texture. As previously stated, the carbide volume fraction represents the experimental factor, and it is assumed to have a fixed effect on the model parameters; instead, the texture is the blocking factor for which a random effect on just the intercept of the model is assumed. These assumptions were confirmed by Figure 11, which indicates that the slope of the relationship between the Voce-model parameters and the carbide volume fraction is independent of the texture, but the intercept does vary with the texture.

**Figure 12:**
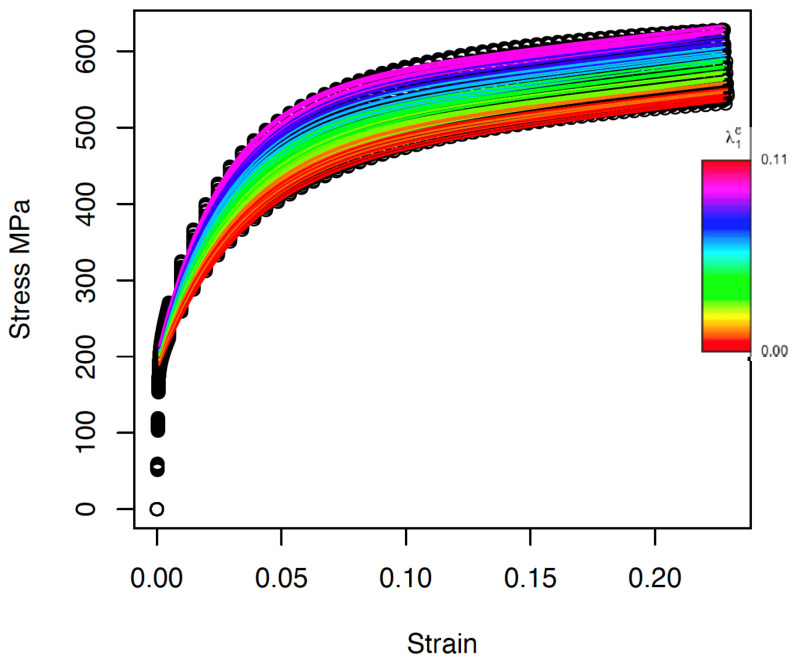
Plot of the estimated stress–strain curve using the Voce model with parameters modeled in terms of the carbide volume fraction and texture in the ferrite phase via linear mixed models.

Using Equation (15), the four models are:

(16) $$\begin{array}{cccccccc} \tau_{0i} & =187.5+\alpha_{0j}+101.4\;\lambda_{1i}^{c}, & \; & \alpha_{0j}∼N\left(0,3.549\right), & \; & i=1,\ldots ,70, & \; & j=1,\ldots ,10\\ \tau_{1i} & =268.3+\alpha_{0j}+621.5\;\lambda_{1i}^{c}, & \; & \alpha_{0j}∼N\left(0,6.033\right), & \; & i=1,\ldots ,70, & \; & j=1,\ldots ,10\\ \theta_{0i} & =8688.5+\alpha_{0j}+42064.6\;\lambda_{1i}^{c}, & \; & \alpha_{0j}∼N\left(0,269.269\right), & \; & i=1,\ldots ,70, & \; & j=1,\ldots ,10\\ \theta_{1i} & =382.9+\alpha_{0j}+205.1\;\lambda_{1i}^{c}, & \; & \alpha_{0j}∼N\left(0,16.617\right), & \; & i=1,\ldots ,70, & \; & j=1,\ldots ,10 \end{array}$$

where all parameters have stress units. Substituting these expressions in the Voce law (Equation (9)) results in the stress–strain curves shown in Figure 12.

The same approach was used for giving a physical meaning to the functional principal component score $A_{1}$ in terms of carbides’ volume fraction and texture. The resulting linear mixed model is:

(17) $$A_{1i}=-1.351+\alpha_{0j}+355.793\;\lambda_{1i}^{c},\;\;\;\;\alpha_{0j}∼N\left(0,3.320\right),\;\;i=1,\ldots ,70,\;\;j=1,\ldots ,10.$$

Combining Equations (12) and (17), the final model for a generic stress–strain curve is then:

(18) $${\hat{\sigma }}_{i}\left(\epsilon \right)={\hat{\sigma }}_{0}\left(\epsilon \right)+\left[-1.351+\alpha_{0j}+355.793\;\lambda_{1i}^{c}\right]\phi_{1}\left(\epsilon ,\right)\;\;\;\;\alpha_{0j}∼N\left(0,3.320\right),$$

**Figure 13:**
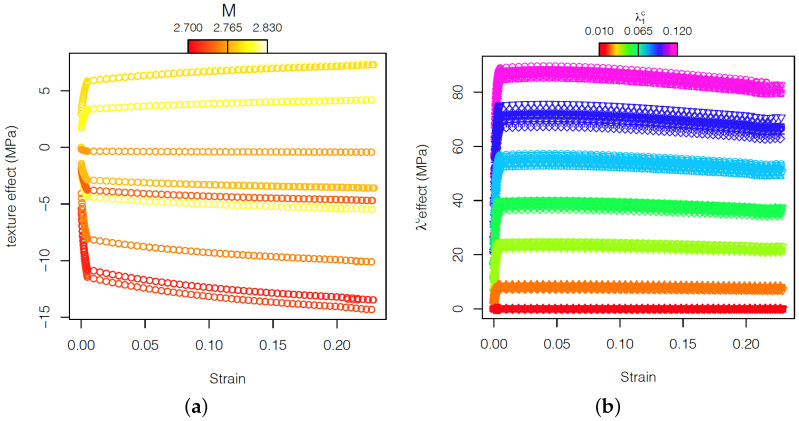
Effect of texture in the ferrite phase (**a**) and of the carbides’ volume fractions (**b**) in the stress–strain curves.

Moreover, multiplying the coefficient of the linear mixed model by the eigenfunction ϕ(ϵ), the effect of the different textures and of the carbides volume fractions at any point of the stress–strain curve can be evaluated (Figure 13a,b).

### 3.1. Goodness of Fit

As a measure of goodness of fit of the two proposed models, the root mean square-error (RMSE) and the mean absolute error (MAE), the most commonly used scale-dependent metrics [52], were computed for every curve and for both models.

**Figure 14:**
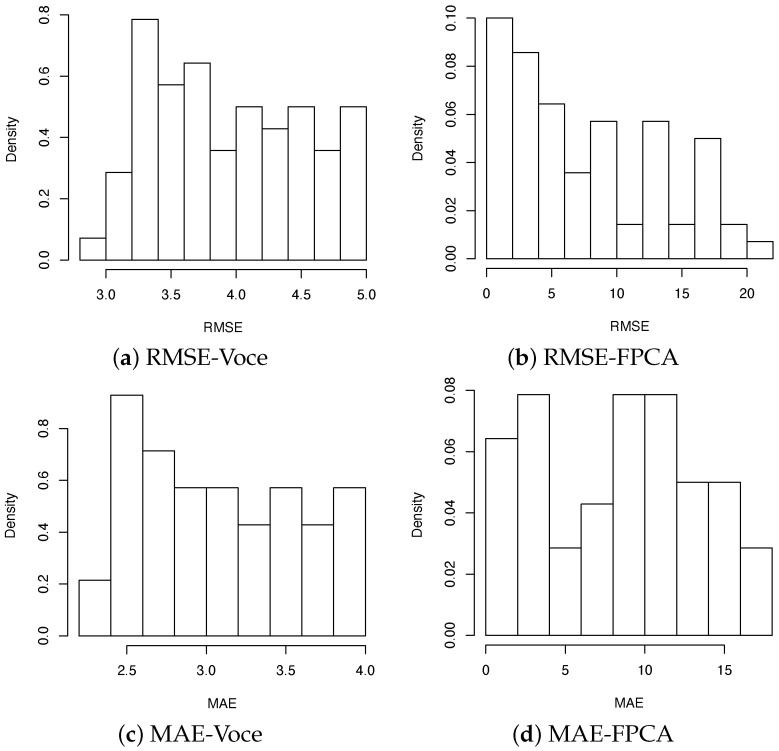
Histograms of the RMSE and the MAE (values in MPa) computed for all curves using the model based on the Voce law (**a**–**c**) and the FPCA approach (**b**–**d**).

The RMSE is the square root of the mean of the square of all errors ($\mathrm{RMSE}=\sqrt{\frac{1}{n}\sum_{i=1}^{N}{\left(y_{i}-\hat{y_{i}}\right)}^{2}}$). The MAE expresses the average model-prediction error in the units of the variable of interest ($\mathrm{MAE}=\frac{1}{n}\sum_{i=1}^{N}\left(\right|y_{i}-\hat{y_{i}}\left|\right)$. Both the RMSE and the MAE are good measures for evaluating the model performance [52], and values close to the random experimental uncertainty indicate good model fitting [53]. Figure 14 shows the goodness-of-fit of the two models and suggests that the two models are reasonably good in representing the tensile behavior of the synthetic microstructures (the order of magnitude of the both the RMSE and the MAE values of the two models can be compared to the overall standard deviation of the stress data that is in the range [12.1; 13.4] MPa representing the intrinsic variability of the data). (Density in Figure 14 is the ratio between the frequency and the width of the class. Note that the scale of the graphs related to the Voce-law approach and the FPCA approach are different.) The ranges of values of both the RMSE and the MAE for the model based on the Voce law are much smaller with respect to the ones based on the FPCA approach. This means that the accuracy of Voce-law fitting is more or less the same for all the textures and the carbide volume fractions considered; on the other hand, the FPCA model is very accurate for some stress–strain curves, but, for most curves, the fitting is not as precise as the Voce model. It should be noted that the model based on the Voce law describes only the plastic strain, whereas the FPCA approach considers the whole-range stress–strain curve.

## 4. Discussion

This work presents a simulation-based approach for investigating the influence of the $M_{23}C_{6}$ carbides on the stress–strain behavior of AISI 420 steel. Using as a starting point geometrical and mechanical information provided in [25], the first step was the generation of synthetic microstructures representing the material under study. Among the models used to describe material microstructures, multi-level Voronoi diagrams prove to be sufficiently flexible for representing the material under study [25]. With this approach, it is possible to control the morphology of the microstructures, modifying one feature at the time, which is not feasible by using experimental thermomechanical treatments. In this specific case, we changed just the carbide volume fraction. For avoiding spurious influences on the microstructure-property relation by the texture, ten different randomly generated textures were considered. In future work, it would be interesting to study the effect of other microstructure features, e.g., placing the carbides at specific locations such as grain boundaries.

Two approaches, the extended Voce model and Functional Principal Component Analysis (FPCA), were followed to understand the influence of carbide fraction on the development of the stress–strain curve controlled by the possible effect of the texture. The aim of this work was not finding the best fitting for the strain–stress curve but illustrating two different methods for obtaining insight into the mechanical behavior of the material using information on microstructure features. This was achieved by the application of a linear mixed model. Voce and other typical models used to describe the material strain–stress curve entail the assumption of a law and then the fitting of parameters. The FPCA approach has the advantage of not requiring any assumption, and it can directly evaluate the influence of an input parameter on the investigated mechanical behavior. In this respect, the approach based on FPCA allows to study the effect of the two microstructural variables, carbides’ volume fraction and texture, at any point of the stress–strain curve, highlighting differences in the intensity of the effect in the different stages of the tensile testing.

The analysis of the linear-mixed model shows that in the Voce model, the carbide volume fraction strongly influences the parameters $\tau_{1}$ and $\theta_{0}$, corresponding to the back-extrapolated critical resolved shear stress (CRSS) and the initial hardening, respectively. Instead, $\tau_{0}$, the initial CRSS, and $\theta_{1}$, the asymptotic hardening rate, are mainly influenced by the texture. In particular, textures with a high Taylor factor present a high value of $\tau_{0}$. The relation with $\theta_{1}$ is less clear, but it indicates that textures with a high Taylor factor correspond to lower values of $\theta_{1}$. In the particular case of the evaluation of the effect of carbide fraction on the strain–stress curve of AISI 420 steel, the potential of FPCA is exemplified in Figure 13. Figure 13 shows that carbides influence more markedly the first part of the plastic flow, from yielding and up to around 0.05 strain, compared to the reference without carbides. In the virtual tensile curves it can be observed that the yield strength increased up to ≈90 MPa, followed by a strong hardening. From 0.05 strain, the effect of carbides became weaker. This behavior was more prominent as the carbide volume fraction increases. That makes the effect of the volume fraction of carbides on the ultimate tensile strength less strong than on the yield stress, causing an increment of at maximum ≈82 MPa corresponding to a 13% carbide volume fraction. These observations are in agreement with the clear influence of the carbides on the initial hardening parameter of the Voce model but not on the asymptotic hardening rate.

The interpretation of the results obtained from Voce and FPCA models are in line with experimental results on an AISI 420 steel with a 0.03 carbide fraction, in which it was demonstrated that the contribution of carbides (and grain boundaries) to the development of heterogeneous local strains decreases with macroscopic strain [25]. It was discussed, in line with Fleck et al. [54], that the observed high hardening rate at low plastic strains originates from a dominant effect of long-range back stresses generated by a misfit between the soft ferrite matrix and the hard $M_{23}C_{6}$ carbides. This misfit is closely related to the development of interface dislocations or geometrically necessary dislocations (GND) at the hard–soft region interfaces. These deformation mechanisms are not explicitly taken into consideration by the crystal plasticity model, and thus there should be another explanation for the results obtained in the Voce and FPCA approaches. In the representative volume elements (RVEs), high internal stresses are developed at ferrite–carbide interfaces at low plastic strain, which are necessary for the simultaneous compatible deformation of hard and soft regions. This fact is shown by Hidalgo et al. [25]. It is reasonable to argue that as the volume fraction of carbides increases, the strain incompatibilities between $M_{23}C_{6}$ carbides and the ferrite matrix increase. Hence, the internal stresses will be higher and so will the effect of carbides on the strain–stress curve as depicted in Figure 13. The local strains in the RVEs become more homogeneous as the plastic strain increases and the grains reorient more favorably for deformation. This suggests that the internal stresses that originated from strain incompatibilities have a smaller effect on the overall macroscopic strain and that the grain orientation also plays a role in deformation.

Figure 13 displays that the influence of texture on the plastic flow obtained from FPCA model, independently of it having positive or negative effect, is stronger as the strain increases. The influence is in general stronger as the texture (read Taylor factor) deviates more from the reference value, although there is not an obvious trend. In any case, textures with a variation in Taylor factor (value corresponding to random texture) cause a slight variation on the yield strength of ≈5 MPa. It is worth noting that the Taylor factor represents the average effect of the orientation of all grains and that small or large dispersion of orientations can lead to a similar Taylor factor. Moreover, the Taylor factor given by the full-constraint Taylor model does not take into account grain-interaction mechanisms. The interaction between grains significantly influences the deformation. The orientations might not be uniformly distributed, and some grain clusters can impair a strong effect on deformation, which is not solely explained by the Taylor factor. Hence, the Taylor factor cannot be expected to be fully representative for the variability of texture, especially in RVEs with a small number of grains. Nevertheless, the kind of variability obtained in the current experiments is considered positive for validating the strengths of the FPCA model.

As pointed out previously, the reader should bear in mind that tensile curves have been created from the application of a phenomenological hardening law in a crystal plasticity model previously applied and validated in [25]. This hardening law is rather simple but has demonstrated to be sufficiently accurate in predicting metals’ behavior upon the application of deformation. Moreover, steels do not typically exhibit a random texture or precipitates with equivalent size as assumed in this work. Despite these artificial limitations introduced in the current study, the proposed simulation set up enabled to prove the advantages of using FPCA approaches for a better understanding of the relation between microstructural and mechanical properties. An interesting, more-advanced application is, instead of directly applying a macroscopic model, to use FPCA to find a hardening law dependent on the carbide volume fraction to use in crystal plasticity models. Such an approach will give better results, since its implementation is more sophisticated. Another interesting application of FPCA is to find the dependence of hardening on non-microstructural parameters, but loading conditions, like the strain rate and the temperature directly from experimental stress–strain curves.

## 5. Conclusions

1. The design of a randomized-block experiment allows to study the contribution of the $M_{23}C_{6}$ carbides on the stress–strain behavior of AISI 420 steel controlling for the possible confounding effect of the textures.
2. Multi-level Voronoi diagrams prove to be a flexible model that allow to represent the microstructure under analysis.
3. The approach is simulation-based, and hence it is fully reproducible and tuneable for other microstructure-features and mechanical-properties investigation.
4. The FPCA model is a flexible approach that does not require any physical assumption and that can be applied also for modeling the mechanical behavior, highlighting the effect of the different sources of variations given by the microstructural features.
5. Linear mixed-effects models are able to give a clear interpretation of the model parameters of both the Voce and the FPCA model in terms of the carbide volume fraction and the textures.
6. The presented research methodology can be applied to other alloys with different precipitates such as graphite in cast iron or intermetallics in superalloys.

## Figures and Tables

**Table 1:** Estimated moments of the geometrical features of 1000 grains obtained by EBSD measurements.

(a) Ferrite		(b) Carbides	
Volume Fraction	0.968	Volume Fraction	0.032
Mean Volume ($\mu m^{3}$)	2.58±0.05	Mean Volume ($\mu m^{3}$)	0.45±0.03
Mean Area ($\mu m^{2}$)	4.43±0.07	Mean Area ($\mu m^{2}$)	0.70±0.03

**Table 2:** Materials parameter for DAMASK model implementation.

Parameter	Unit	Ferrite	$M_{23}C_{6}$ Carbides
$C_{11}$	GPa	233	550.8
$C_{12}$	GPa	135	225.9
$C_{44}$	GPa	128	140
${\dot{\gamma }}_{0}$	s${}^{-1}$	0.001	0.001
$n_{slip}$	-	10	200
$\tau_{C}^{\eta }$	MPa	80	1600
$\tau_{sat}$	MPa	250	1800
$h_{0}$	MPa	549.4	20,000
*a*	-	2.25	1.1

## Data Availability

The raw data and processed data required to reproduce these findings are available to download from https://github.com/martinavitt/A-data-driven-approach-for-studying-the-influence-of-carbides-on-work-hardening-of-steel.

## Abbreviations

The following abbreviations are used in this manuscript:

FPCA	Functional Principal Component Analysis
DAMASK	Düsseldorf Advanced Material Simulation Toolkit
ODF	Orientation Distribution Function
RVE	Representative Volume Element
FC	Full Constraints
FFT	Fast Fourier Transform
RMSE	Root Mean Square Error
MAE	Mean Absolute Error
